# Evaluation of Toxic Amyloid β42 Oligomers in Rat Primary Cerebral Cortex Cells and Human iPS-derived Neurons Treated with 10-Me-Aplog-1, a New PKC Activator

**DOI:** 10.3390/ijms21041179

**Published:** 2020-02-11

**Authors:** Kazuma Murakami, Mayuko Yoshimura, Shota Nakagawa, Toshiaki Kume, Takayuki Kondo, Haruhisa Inoue, Kazuhiro Irie

**Affiliations:** 1 Division of Food Science and Biotechnology, Graduate School of Agriculture, Kyoto University, Kyoto 606-8502, Japan; myk0128dw@gmail.com; 2 Department of Pharmacology, Graduate School of Pharmaceutical Sciences, Kyoto University, Kyoto 606-8501, Japan; nakagawa.shota.0812@gmail.com (S.N.); tkume@pha.u-toyama.ac.jp (T.K.); 3 Department of Applied Pharmacology, Graduate School of Medicine and Pharmaceutical Sciences, University of Toyama, Toyama 930-0194, Japan; 4 Center for iPS Cell Research and Application (CiRA), Kyoto University, Kyoto 606-8507, Japan; takayuki.kondo@cira.kyoto-u.ac.jp (T.K.); haruhisa@cira.kyoto-u.ac.jp (H.I.); 5 iPSC-based Drug Discovery and Development Team, RIKEN BioResource Research Center (BRC), Kyoto 619-0237, Japan; 6 Medical-risk Avoidance based on iPS Cells Team, RIKEN Center for Advanced Intelligence Project (AIP), Kyoto 606-8507, Japan

**Keywords:** alzheimer’s disease, amyloid *β*, bryostatin-1, ECE1, iPS, nELAV, neurotoxicity, oligomer, protein kinase C, *α*-secretase

## Abstract

Amyloid *β*42 (Aβ42), a causative agent of Alzheimer’s disease (AD), is derived extracellularly from A*β* precursor protein (APP) following the latter’s cleavage by *β*-secretase, but not α-secretase. Protein kinase C*α* (PKC*α*) activation is known to increase *α*-secretase activity, thereby suppressing A*β* production. Since Aβ42 oligomer formation causes potent neurotoxicity, APP modulation by PKC ligands is a promising strategy for AD treatment. Although bryostatin-1 (bryo-1) is a leading compound for this strategy, its limited natural availability and the difficulty of its total synthesis impedes further research. To address this limitation, Irie and colleagues have developed a new PKC activator with few side effects, 10-Me-Aplog-1, (**1**), which decreased Aβ42 in the conditioned medium of rat primary cerebral cortex cells. These results are associated with increased α-secretase but not PKC*ε*-dependent A*β*-degrading enzyme. The amount of neuronal embryonic lethal abnormal vision (nELAV), a known *β*-secretase stabilizer, was reduced by treatment with **1**. Notably, **1** prevented the formation of intracellular toxic oligomers. Furthermore, **1** suppressed toxic oligomerization within human iPS-derived neurons such as bryo-1. Given that **1** was not neurotoxic toward either cell line, these findings suggest that **1** is a potential drug lead for AD therapy.

## 1. Introduction

The 40-mer and 42-mer amyloid *β*-proteins (Aβ40 and Aβ42) are considered causative agents of Alzheimer’s disease (AD) [1,2]. Aβ40 and Aβ42 are known to be produced from Aβ precursor protein (APP) following cleavage of the latter by *β*-secretase, but not α-secretase. APP proteolysis may be more complex, given the recent discovery of APP proteolysis by *η*- and *δ*-secretases, for example, in [3]. The ability of Aβ42 to aggregate and exhibit neurotoxicity is higher than that of Aβ40 despite the lower in vivo amounts of Aβ42 [4]. Aβ42 oligomer formation causes synaptic dysfunction and neuronal death in AD pathology, whereas the contribution of end-stage mature fibrils of Aβ42 to AD is lower than that of oligomers [5]. Higher-order toxic oligomers that show potent synaptotoxicity and neurotoxicity have been reported, such as protofibrils (PFs), Aβ-derived diffusible ligands, and amylospheroids [6]. Therefore, suppressing toxic oligomerization of Aβ42 is a favorable strategy for developing AD therapies. This suppression can also be achieved by simultaneously decreasing Aβ production while inducing Aβ degradation.

Protein kinase C (PKC) is a family of serine/threonine kinases that plays a pivotal role in various biological events such as signal transduction, proliferation, and apoptosis mediated by the second messenger 1,2-diacyl-*sn*-glycerol [7]. The PKC family, which contains at least 10 isozymes, is divided into three groups, namely conventional (*α*, *β*I, *β*II, and *γ*), novel (*δ*, *ε*, *η*, and *θ*), and atypical (*μ*, *ξ*, and *ι*) [7]. PKC activity is related to memory formation and learning [8], while PKC downregulation may induce cognitive impairment and memory loss in AD [9]. Regarding Aβ-driven molecular events, PKC*α* reportedly upregulates *α*-secretase activity either directly or indirectly through the mitogen-activated protein kinase (MAPK) pathway [10]. PKC*α* activation in a mouse model of AD has beneficial effects on AD pathology, including the disruption of Aβ production and reduction of toxic A*β* oligomer formation [11]. Neuronal embryonic lethal abnormal vision (nELAV), also known as HuD protein, may contribute to mRNA stability through a PKC*α*-dependent mechanism due to adenine- and uridine-rich elements (AREs) [12]. PKC*ε* may also be a target beneficial for preventing AD. A mouse study demonstrated that PKCε activation reduces senile plaque formation, although its effect on oligomer generation was not determined [13]. Similarly, the stimulator specific for PKC*ε* (DCP-LA) rescued synaptic dysfunction and cognitive deficits as well as senile plaques in another mouse study [14]. PKCε stimulates the degradation of Aβ42 and Aβ40 by activating endothelin converting enzyme 1 (ECE1) [15]. These reports indicate that PKC activation may offer a promising strategy for AD treatment.

**Figure 1:**
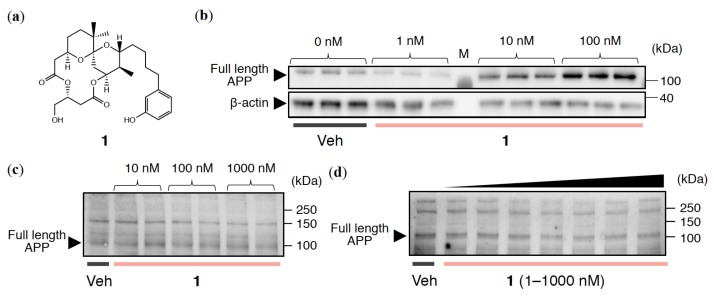
(**a**) Structure of 10-Me-Aplog-1 (**1**). APP expression levels in (**b**) HEK293-APPwt, (**c**) SH-SY5Y, and (**d**) rat primary cerebral cortex cells treated with **1** at the indicated concentrations for 24 h. M indicates marker. In (**d**), 1, 10, 50, 100, 500, and 1000 nM (from left to right) of **1** were used. Veh: vehicle.

Bryostatin-1 (bryo-1), which was isolated from the marine bryozoan *Bugula neritina* [16], is a potent PKC activator with few side effects such as tumor-promoting and proinflammatory activities. Bryo-1 was found to activate both PKC*α* and PKC*ε*, and to restore loss of hippocampal synapses and memory impairment by suppressing the levels of Aβ oligomers detected by the A11 antibody [14]. Bryo-1 may have beneficial effects against Aβ-induced abnormality in human fibroblasts [17]. These findings indicate that bryo-1 is a potential drug lead for AD [18]. However, its limited availability from natural sources and the difficulty of total synthesis both hamper further development, despite scalable synthetic routes reported by the Wender [19] and Trost groups [20]. Taking an alternative approach, Irie and colleagues developed 10-Me-Aplog-1 (**1**; Figure 1a), a simplified analog of aplysiatoxin [21], which is a potent PKC activator with tumor-promoting activity. It should be noted that **1** exhibited anti-proliferative activity towards cancer cell lines without significant tumor-promoting or proinflammatory activities [22,23]. 

The ratio of Aβ42 to Aβ40 (Aβ42/Aβ40) is a known biomarker for predicting AD onset in cerebrospinal fluid (CSF) and plasma [24]. However, such a biomarker could correlate with senile plaque depositions containing less toxic fibrils according to brain imaging of Aβ deposition with positron emission tomography (A*β*-PET) [25,26,27]. Furthermore, the PKC activation strategy is not expected to modulate Aβ42/Aβ40, since the proteolysis of APP by *γ*-secretase can predominantly determine the length of secreted Aβ. 

Irie and colleagues identified a toxic Aβ42 conformer with a turn at positions 22–23 (toxic turn) [28], and proposed the ratio of the toxic conformer to total Aβ42 as a possible biomarker for AD progression in CSF using sandwich ELISA specific for Aβ42 toxic oligomers based on the anti-toxic turn antibody (24B3) [29]. A change in Aβ42 toxic conformer ratio may be a good predictor for long-term cognitive outcomes in idiopathic normal pressure hydrocephalus (iNPH) [30]. Toxic conformers can easily form toxic oligomers [31]. Here, we offer a novel, direct evaluation platform that determines the ratio of toxic oligomers to Aβ42 (toxic oligomers/Aβ42) in rat primary cerebral cortex cells and human induced pluripotent stem (iPS)-derived neurons using 24B3-based ELISA [29], which were treated with **1**. The therapeutic potential of **1** and its mechanism of action in AD prevention were also investigated. 

## 2. Results

### 2.1. APP Expression Levels in Cultured Neuronal Cell Lines Treated with 1

The reason why research on PKC modulators faces difficulties in the AD field is the abnormal enhancement of APP itself upon addition of PKC ligand to cultured animal cells, including rat PC12 cells [32] and human HeLa cells [33], resulting in unwanted A*β* overproduction. Alternatively, APP secreted after *α*-secretase processing (sAPP*α*) or AD-index calculated from Erk1/2 phosphorylation have been used as evaluation criteria for PKC modulators [17]; however, there are very few reports concerning the direct quantification of Aβ in cell-based experiments. As expected, **1** enhanced APP levels in HEK293 cells overexpressing wild-type APP (HEK293-APPwt) in a dose-dependent manner (Figure 1b). By contrast, APP levels in both SH-SY5Y cells (Figure 1c) and rat primary cerebral cortex cells (Figure 1d) were largely unaltered. 

### 2.2. Effects of 1 on Extracellular Aβ42/Aβ40 and Aβ Oligomerization in Rat Primary Cerebral Cortex Cells

**Figure 2:**
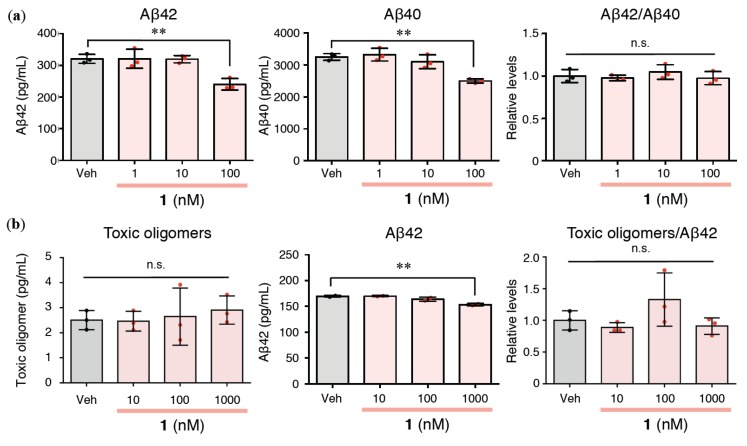
(**a**) Monomeric Aβ42, Aβ40, and their ratio (Aβ42/Aβ40) in the conditioned medium of rat primary cerebral cortex cells treated with **1** at the indicated concentrations for 24 h. (**b**) Toxic Aβ oligomers, monomeric Aβ42, and their ratio (toxic oligomers/Aβ42) in the conditioned medium of rat primary cerebral cortex cells treated with **1** at the indicated concentrations for 6 h. The data are presented as mean ± SD (*n* = 3). ^**^p < 0.01 versus Veh (vehicle). n.s.: not significant. Red or black dots represent each value.

Since the amount of Aβ42 secreted by SH-SY5Y cells was near to the detection limit of specific ELISA (#27711 Human Amyloid *β* 1-42 Assay Kit—IBL), we selected rat primary cerebral cortex cells for evaluating PKC modulators in the following study. After a 24 h incubation, **1** did not reduce Aβ42/Aβ40 as expected above, because the amounts of both Aβ42 and Aβ40 were lowered (Figure 2a). 12-*O*-Tetradecanoylphorbol 13-acetate (TPA) is a PKC ligand that exerts a similar effect [34]. Because the extracellular levels of toxic oligomers after a 24 h incubation were under the detection limit for specific ELISA (#27709 Human Amyloid *β* Toxic Oligomer Assay Kit—IBL) and Aβ42 easily aggregates to form amyloid fibrils after a 24 h incubation in vitro [35,36], we sampled at an earlier time point, 6 h, to determine the formation of toxic Aβ oligomers. As shown in Figure 2b, the ratio of toxic oligomers to Aβ42 (toxic oligomers/Aβ42) in cerebral cortex cells did not increase following treatment with **1** even at a higher concentration range than that in Figure 2a. However, the toxic oligomer levels were unchanged by **1** (Figure 2b).

### 2.3. Effects of 1 on Aβ Production and Degradation in Rat Primary Cerebral Cortex Cells

**Figure 3:**
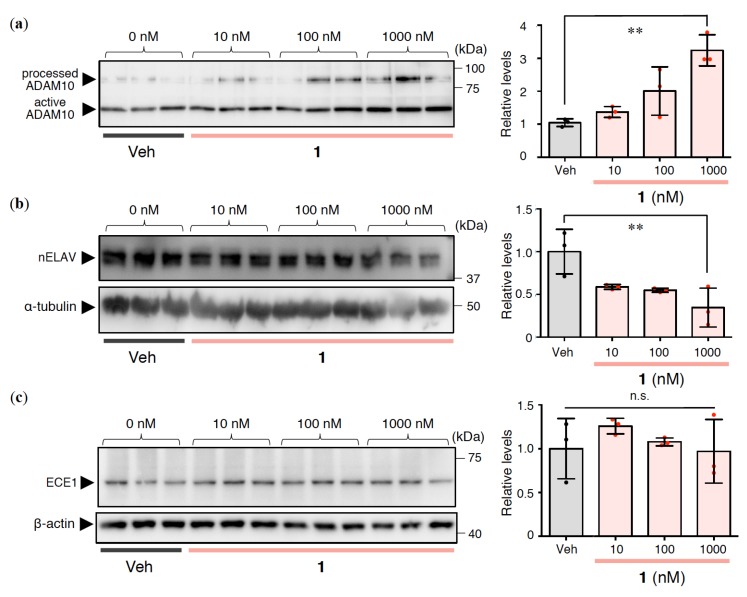
(**a**) Processed ADAM10, (**b**) nELAV, and (**c**) ECE1 in the cell lysate prepared from rat primary cerebral cortex cells treated with **1** at indicated concentrations for 24 h. The relative levels of (**a**) active ADAM10, (**b**) *α*-tubulin, and (**c**) *β*-actin are presented as mean ± SD (*n* = 3). ^**^p < 0.01 versus Veh (vehicle). n.s.: not significant. Red or black dots represent each value.

Given the moderate reduction in Aβ42 secretion to the extracellular space caused by **1** treatment (Figure 2b), we investigated the contribution of **1** to Aβ production and degradation in cultured cells. The concentration of **1** was set to 10–1000 nM in the following study of primary cultured cells. The amount of disintegrin and metalloproteinase 10 (ADAM10), as one of the α-secretases, was increased in Western blotting, using the ratio of the processed to active form of ADAM10 in the case of **1** (Figure 3a). nELAV proteins are known to act as PKC*α*-dependent Aβ modulators via *α*-secretase [12,37] or *β*-secretase [38]. As shown in Figure 3b, the amounts of nELAV were decreased by **1**.

Next, ECE1 levels were also measured. ECE1 levels were almost unchanged in cells treated with **1** (Figure 3c). These results indicate that the decrease in Aβ42 caused by **1** could be due to enhanced α-secretase expression, but not Aβ degradation.

### 2.4. Effects of 1 on Intracellular Aβ Oligomerization in Rat Primary Cerebral Cortex Cells

**Figure 4:**
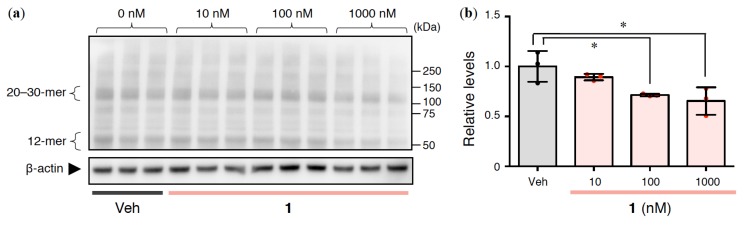
(**a**,**b**) Toxic oligomer formation in lysate from rat primary cerebral cortex cells treated with **1** at the indicated concentration for 6 h. (**a**) The representative Western blot shown was probed with anti-Aβ42 toxic turn (24B3) antibody. (**b**) Band intensities corresponding to 20–30-mers relative to *β*-actin in (**a**) are presented as mean ± SD (*n* = 3). ^*^p < 0.05 versus Veh (vehicle). Red or black dots represent each value.

Intracellular Aβ accumulation appears to be an early event in AD pathogenesis. In particular, Aβ oligomerization may begin to induce mitochondrial toxicity, proteasome impairment, and synaptic damage [39]. To elucidate the intracellular mechanism, lysates were prepared from cells after 6 h of incubation with **1** and subjected to Western blotting using 24B3 antibody [29]. Notably, the formation of intracellular toxic oligomers, which are 20–30-mers according to synthetic studies [40,41] of Aβ oligomer models that inhibited long-term potentiation (LTP) in mouse hippocampal slices (T. Kume, personal communication, unpublished results), was significantly decreased by **1** (Figure 4). These results suggest that **1** may modulate toxic Aβ oligomerization.

### 2.5. Effects of 1 on the Cytotoxicity of Rat Primary Cerebral Cortex Cells

**Figure 5:**
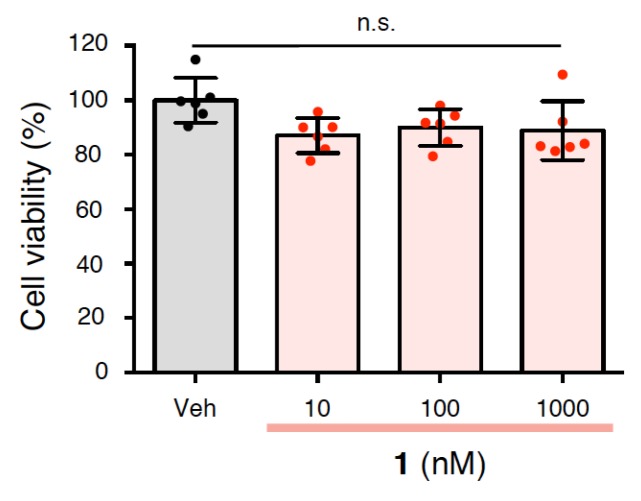
Neurotoxicity of rat primary cerebral cortex cells treated with **1** at the indicated concentrations for 24 h. The data are presented as mean ± SD (*n* = 6). n.s.: not significant. Veh: vehicle. Red or black dots represent each value.

To examine the neurotoxicity of **1**, a 3-(4,5-dimethylthiazol-2-yl)-2,5-diphenyltetrazolium bromide (MTT) assay was performed on rat primary cerebral cortex cells. As shown in Figure 5, it was confirmed that **1** did not exhibit neurotoxicity at the concentrations used in the above tests (Figure 2b,3,4). This finding suggests that **1** is potentially as safe as bryo-1, with few side effects.

### 2.6. Effects of 1 on Aβ42/Aβ40, Aβ Oligomerization, and Neurotoxicity in Human iPS-Derived Neurons

**Figure 6:**
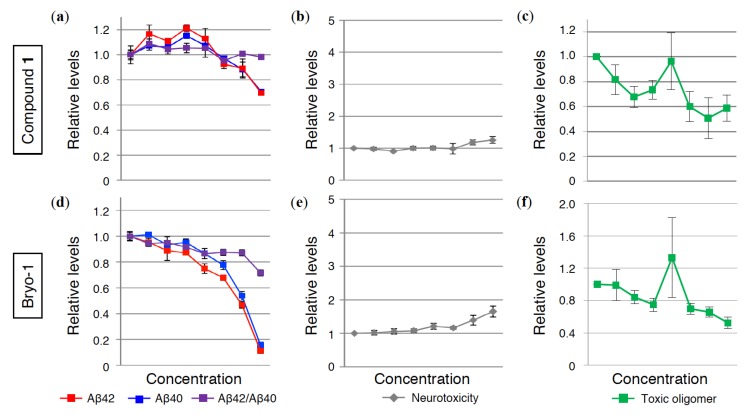
(**a**,**d**) Monomeric Aβ42, Aβ40, and Aβ42/Aβ40 in conditioned medium, (**b**,**e**) neurotoxicity, and (**c**,**f**) toxic Aβ42 oligomers in lysate from human iPS-derived neurons treated with (**a**,**b**,**c**) **1** and (**d**,**e**,**f**) bryo-1 at the indicated concentration (0, 1, 3, 10, 30, 100, 300, and 1000 nM from left to right) for 24 h. The data are presented as mean ± SEM (*n* = 3).

To further verify the preventative effects of **1** against AD, human iPS-derived neurons were adopted for this experiment because of a slight difference in Aβ sequence between rat and human. Recent studies also imply a large gap in the effectiveness of drug discovery studies between iPS-derived neurons and cultured cell lines [42]. Recently, Inoue and colleagues developed a reliable and robust iPS-based screening system for anti-Aβ drugs [43]. After incubating the differentiated neurons from iPS with PKC ligands for 24 h, Aβ42 and Aβ40 levels in the conditioned medium were calculated using electrochemiluminescence assays. Bryo-1 was used as a positive control, which significantly decreased the amount of Aβ42 and Aβ40 in a dose-dependent manner. Bryo-1 therefore suppressed the Aβ42/Aβ40 ratio (Figure 6d). Treatment with **1** lowered Aβ42 and Aβ40 levels to almost the same extent, resulting in almost no alternation of Aβ42/Aβ40 (Figure 6a). **1** failed to show cytotoxicity such as bryo-1 (Figure 6b,e) measured by the ToxiLight assay that reflects the release of adenylate kinase from damaged cells [44].

Lysate prepared from iPS-derived neurons was subjected to ELISA measurement for toxic oligomers (Figure 6c,f). In Figure 6c, the amount of Aβ42 toxic oligomers following **1** treatment showed a tendency to decrease, like bryo-1, in a dose-dependent manner (Figure 6f), in spite of one anomalous value at 30 nM, which might originate from a technical issue. These findings suggest that **1** may also prevent toxic oligomer formation in iPS-derived neurons.

## 3. Discussion

Alkon and colleagues hypothesized that deficits in PKC signaling are involved in AD symptoms [18]. PKC*α* and PKC*ε* are thought to induce Aβ diminution, leading to beneficial effects for AD. Indeed, the results of several clinical trials provide encouragement for bryo-1 as a potential drug against AD [45]. It is worth noting that **1** prevented nELAV accumulation within the cell (Figure 3b). nELAV levels were higher in AD patients compared with non-AD controls [38]. The nELAV-driven stabilization of *β*-secretase mRNA (*β*-site amyloid *β* precursor protein cleaving enzyme, BACE1) [38] and tau mRNA [46] may be involved in AD progression. On the other hand, experiments using SH-SY5Y cells suggest that the stabilization of ADAM10 by the binding of nELAV may contribute to beneficial effects against AD via the PKC*α* pathway [12]. Bryo-1 counteracted the deficit in ADAM10 in SH-SY5Y cells in which HuD expression had been silenced [47]. Although the involvement of nELAV in AD remains controversial, nELAV is a novel putative target for anti-AD therapies. Furthermore, Jarosz-Griffiths et al. reported that ADAM10-modulated shedding of cellular prion protein reduced the neurotoxicity of Aβ oligomers [48]. The present findings illustrate that **1** prevented the formation of intracellular Aβ42 oligomers as well as extracellular Aβ42, which is associated with enhanced α-secretase cleavage of APP. Further studies will be required to clarify whether **1** might affect toxic oligomerization directly, and if so, how. Given that the parent analogue (Aplog-1) of **1** can activate PKCδ [22] and **1** binds potently to the PKC*α*-C1A and PKC*ε*-C1B domains [23], **1** is a promising substitute for bryo-1 as a therapeutic drug lead for AD.

Recently, Yanagisawa and colleagues identified plasma APP669-711/Aβ42 [49] in addition to Aβ42/Aβ40 as an alternative biomarker using Japanese and Australian cohorts [50]. Using a composite biomarker calculated from APP669-711/Aβ42 and Aβ42/Aβ40 may enhance the accuracy of diagnosis during disease progression from mild cognitive impairment (MCI) to AD. However, in their work, the potential of Aβ oligomerization in CSF or plasma as a biomarker was not fully addressed. Recently, a detection method for Aβ oligomers using single molecule arrays (Simoa) as a highly sensitive platform was reported using the same anti-Aβ N-terminal antibody (bapineuzumab), both for antigen capture and detection [51]. However, this strategy cannot exclude the possibility of detecting mature fibrils, resulting in lower specificity for Aβ oligomers [52]. The use of the anti-N-terminal antibody (82E1) [53] may address the problem by using the same antibodies for capture and detection [54]. Alternatively, the development of highly specific antibodies for toxic oligomeric species with synaptotoxicity would be most ideal for finding biomarkers. In the Aβ42 toxic oligomer ELISA used in this study, the 24B3 antibody against the Aβ toxic turn and 82E1 antibody against the Aβ N-terminus are used for detection and capture, respectively [29].

Ohshima et al. reported that familial mutations of AD increase oligomer formation of Aβ in the conditioned medium of wild-type APP-transfected cells, but intracellular levels of Aβ oligomer in these mutant APP-transfected cells were unaltered compared with wild-type APP-transfected cells [55]. These results may be due to the stronger ability of Aβ either to be formed or to aggregate due to these mutations in the precursor APP, namely Swedish, Dutch, and London mutations. The Osaka mutant (E693Δ) of Aβ tends to be found as oligomers within cell bodies in both cultured cells [56] and human iPS-derived neurons [57]. It was therefore difficult to determine oligomer levels in non-mutated APP cell models with precision. Regarding the intracellular accumulation of Aβ, the key question of how intracellular Aβ accumulates remains unanswered, thereby invoking the involvement of tau pathology; that is, the possible interaction of intraneuronal Aβ with neurofibrillary tangles [58]. The relevance of liquid–liquid phase separation to intracellular accumulation of amyloidogenic proteins (tau [59,60] and TDP-43 [61]) should also be considered.

In conclusion, to the best of our knowledge, we have developed the first direct evaluation system not only for Aβ monomers, but also for their assembly into toxic oligomers in small amounts using two reliable and prevalent cell models of AD. Compared with bryo-1, whose efficiency has been recognized in several clinical trials for AD and cancer, **1** may play a pivotal role in AD prevention as a promising drug lead.

## 4. Materials and Methods 

### 4.1. Rat Primary Cerebral Cortex Cells

Animals were treated according to guidelines issued by the Kyoto University Animal Experimentation Committee and by the Japanese Pharmacological Society. The experimental procedures were approved by the Kyoto University Animal Experimentation Committee [#16-12-1 (14 Mar 2016), #16-12-2 (21 Mar 2017)]. Primary cultures were obtained from the cerebral cortex of fetal Wistar rats (Nihon SLC; 17–19 d of gestation) as previously described [62]. Briefly, single cells dissociated from whole cerebral cortices of fetal rats were plated on 0.1% polyethyleneimine-coated plastic 12-well plates (10^6^ cells/well, 1 mL). Cells were incubated in Eagle’s minimal essential medium (E-MEM) supplemented with 10% heat-inactivated fetal bovine serum (FBS) before half the medium was exchanged for fresh medium 2 and 4 d after plating. Subsequently, half the medium was exchanged for fresh medium containing 20 nM cytosine arabinoside 6 d after plating and again with fresh medium containing 10% heat-inactivated horse serum (HS) 8 d after plating. The cultures were maintained at 37 °C under a humidified 5% CO_2_ atmosphere. Mature cerebral cortex cell cultures (10 d after plating) were used for all experiments. 

DMSO stock of **1** was dissolved in E-MEM with 10% heat-inactivated HS (the concentration of DMSO in the medium was under 0.1%). After 6 or 24 h incubation, 100 μL of cell lysis buffer (RIPA buffer, Wako, Tokyo, Japan) containing a phosphatase inhibitor cocktail (Roche, Mannheim, Germany) and protease inhibitor cocktail (Roche) was added to prepare cell lysates. Supernatant was obtained by centrifugation (17,860 *g*, 4 °C) and stored at –80 °C until use. 

### 4.2. ELISA

To determine the amounts of Aβ42 [#290-62601 Human/Rat β Amyloid(42) ELISA Kit Wako (Osaka, Japan) or #27711 Human Amyloid β 1-42 Assay Kit—IBL (Gunma, Japan)] and Aβ toxic oligomers (#27709 Human Amyloid β Toxic Oligomer Assay Kit—IBL), 100 μL of cell lysate was applied to the corresponding sandwich ELISA plate.

### 4.3. Western Blotting

Total protein concentration of the brain was determined using the Bradford protein assay (Bio-Rad; Hercules, CA, USA). Brain proteins diluted to 1 μg/μL were treated with 4× LDS sample buffer (Invitrogen; Carlsbad, CA, USA) and 5 mM dithiothreitol before heating at 70 °C for 10 min. The denatured sample solution was subjected to Western blotting, following SDS-PAGE on a 10% Bis-Tris gel (Invitrogen) and subsequent transfer to PVDF (0.22 μm pore size, Bio-Rad). PVDF membranes were blocked in 2.5% ECL prime blocking (GE Healthcare; Madison, WI, USA) dissolved in phosphate-buffered saline (PBS) containing 0.5% Tween-20 (PBS-T), and incubated with primary antibody at the following dilutions: 1:1000 anti-Aβ (4G8) (Signet; Dedham, MA, USA), 1:1000 anti-APP(N) (IBL), 1:1000 anti-Aβ42 toxic turn (24B3) (IBL, Gunma, Japan), 1:500 anti-ADAM10 (B-3) (IBL, Gunma, Japan), 1:140 anti-nELAV(HuD+HuC) (Santa Cruz; Santa Cruz, CA, USA), or 1:1000 anti-ECE1 (abcam; Cambridge, MA, USA). Following primary antibody incubation, blots were washed before being incubated with the appropriate secondary antibody. Blots were developed with enhanced chemiluminescence and quantified using Lumino Graph II (ATTO; Tokyo, Japan).

### 4.4. MTT Assay

Neurotoxicity was assessed by the MTT assay according to a previously reported protocol [63]. In brief, mature cerebral cortex cultures were moved to Neurobasal Medium with 2% B-27 supplement, 25 μM sodium glutamate, and penicillin/streptomycin before plating on 96-well plates (1.5 × 10^6^ cells/well, 100 μL). Four days after plating, the medium was replaced with sodium glutamate-free Neurobasal Medium. Half the medium was exchanged for fresh medium 7 or 8 d after plating, and all the medium was exchanged for Neurobasal Medium with 2% B-27 supplement minus AO (Gibco; Grand Island, NY, USA), and penicillin/streptomycin containing DMSO stock of **1** (10 d after plating). After incubation for 24 h, the culture medium was replaced with a medium containing 0.5 mg/mL MTT, and cells were incubated for 15 min at 37 °C. After removing the medium, 2-propanol (100 μL) was added to lyse the cells, and absorbance was measured at 595 nm with an absorption spectrophotometer (MultiScan JX, Thermo Scientific; Waltham, MA, USA). The absorbance measured following vehicle treatment (DMSO final concentration = 0.1%) was fixed as 100% for comparison.

### 4.5. Generation and Characterization of Human iPS-Derived Neurons

Human iPS cells of Alzheimer’s patients were generated as described from skin fibroblasts [43,57] and maintained using StemFit AK02N medium (Ajinomoto, Tokyo, Japan) [64] and expanded for neural differentiation. To establish a robust and rapid differentiation method, we utilized direct conversion technology. We differentiated iPS cells into neurons by using a direct conversion method, as previously described [43]. Briefly, we transduced human NGN2 cDNA by using the piggyBac transposon system, transiently under tetracycline-inducible promoter (tetO), and converted iPS cells into neuronal cells with more than 96% purity.

### 4.6. Electrochemiluminescence Assays

Aβ species in culture media after 24 h cultivation with PKC ligands were measured by human (6E10) Aβ 3-VPlex Kit (Meso Scale Discovery; Rockville, MD, USA). This assay uses the 6E10 anti-*β*-amyloid antibody to capture Aβ peptides and SULFO-TAG-labeled C-terminus specific anti-Aβ antibodies for detection by electrochemiluminescence with Sector Imager 2400 (Meso Scale Discovery). Quantified Aβ values were adjusted using total protein in neurons and compared among conditions.

### 4.7. ToxiLight Assay

Cytotoxicity was determined to measure the release of the enzyme adenylate kinase from damaged cells [44] using The ToxiLight^TM^ Non-destructive Cytotoxicity BioAssay Kit (Lonza; Walkersville, MD, USA). In brief, the cultured medium was collected after 24 h incubation, and applied to the assay.

### 4.8. Statistical Analyses

The differences were subjected to one-way analysis of variance (ANOVA) followed by Bonferroni’s test; *p* values < 0.05 versus vehicle were considered significant.

## Abbreviations

Aβ	Amyloid *β*-protein
AD	Alzheimer’s disease
ADAM10	A disintegrin and metalloproteinase 10
APP	Amyloid *β* precursor protein
BACE1	*β*-site amyloid *β* precursor protein cleaving enzyme 1
DMSO	Dimethyl sulfoxide
ECE1	Endothelin converting enzyme 1
ELISA	Enzyme-linked immunosorbent assay
E-MEM	Eagle’s minimal essential medium
FBS	Fetal bovine serum
HFIP	1,1,1,3,3,3-hexafluoro-2-propanol
HRP	Horseradish peroxidase
HS	Horse serum
iPS	Induced pluripotent stem
MCI	Mild cognitive impairment
MTT	3-(4,5-dimethylthiazol-2-yl)-2,5-diphenyltetrazolium bromide
PBS	Phosphate-buffered saline
PKC	Protein kinase C
sAPP	Secreted amyloid *β* precursor protein
WB	Western blotting
